# The Impact of Nutritional Knowledge of Mothers on Their Children’s Nutritional Knowledge and Weight Status †

**DOI:** 10.3390/healthcare13172226

**Published:** 2025-09-05

**Authors:** Mai Adil Ghabashi, Abrar M. Babateen, Alyaa M. Zagzoog, Abeer M. Aljaadi

**Affiliations:** 1 Clinical Nutrition Department, Faculty of Applied Medical Sciences, Umm Al-Qura University, P.O. Box 715, Makkah 21955, Saudi Arabia; ambabteen@uqu.edu.sa (A.M.B.); amjaadi@uqu.edu.sa (A.M.A.); 2 Department of Community Health, Faculty of Applied Medical Sciences, Northern Border University, P.O. Box 715, Arar 91431, Saudi Arabia; alyaa.zagzoog@nbu.edu.sa

**Keywords:** nutrition knowledge, children, mothers, malnutrition, BMI, obesity, fruit and vegetable consumption, dietary habits

## Abstract

**Objectives:** This cross-sectional study assessed the nutritional knowledge of Saudi mothers and their children. Then, it examined the association between the nutritional knowledge of mothers and the nutritional knowledge and weight status of their schoolchildren in Makkah City, Saudi Arabia. **Methods:** The mothers’ nutritional knowledge was assessed using the validated Arabic version of the General Nutrition Knowledge Questionnaire (GNKQ). The children’s nutritional knowledge was assessed through an interviewer-administered questionnaire, and their weight status was determined based on their Body Mass Index (BMI) Z-score, which was calculated according to their anthropometric measurements. **Results:** One hundred children and sixty mothers were included in this study. Only 6.67% of the mothers had a high level of nutrition knowledge. A total of 54% of their children had low nutritional knowledge scores, and approximately 27% was classified as having excess weight. More than 40% of the children reported consuming fruits and vegetables 4–5 times/week, whereas 50% of the children consumed fast foods 1–3 times/week. Multiple linear regression analyses showed that higher nutritional knowledge scores among the children were significantly associated with higher mothers’ knowledge scores [(0.06 (95%CI: 0.0.03, 0.0.08)] and older age among these children [0.61 (95%CI: 0.44, 0.77)], as the older children had higher knowledge scores. The children’s nutritional knowledge, however, was not associated with the child’s sex, mothers’ age, or mothers’ education. The maternal knowledge scores were not associated with the child’s weight status. **Conclusions:** Given that the nutrition knowledge scores of Saudi mothers are significantly associated with those of their children, but not with their weight status, it can be concluded that nutrition knowledge alone may not be sufficient to address the overweight and obesity epidemic in Saudi Arabia. However, it remains a crucial component of multifaceted interventions that also enhance physical activity and promote behavior change to improve health outcomes and weight status in the Saudi population.

## 1. Significance

Poor nutritional knowledge among mothers adversely affects the health and well-being of schoolchildren. Saudi Arabia has a high rate of childhood obesity. In Saudi Arabia, however, research on the relationship between mothers’ nutritional knowledge and their children’s knowledge and nutritional status is limited. More specifically, regional-level data remain limited, particularly in cities like Makkah, which has unique demographic and lifestyle characteristics that may influence dietary patterns and health behaviors. As the spiritual capital of the Islamic world, Makkah attracts a diverse and transient population throughout the year, especially during pilgrimage seasons. This unique demographic composition, combined with rapid urbanization, shifting dietary practices, and increased exposure to global food markets, may contribute to distinct nutritional challenges that are not seen in other regions in the Kingdom. This study is among the first to explore the associations between maternal nutritional knowledge and the dietary habits of schoolchildren in Makkah City. The findings indicated that the nutritional knowledge of Saudi schoolchildren was impacted by their mothers’ nutritional knowledge. To enhance the nutritional knowledge and dietary habits of Saudi children, it is essential to improve their mothers’ understanding of nutrition through multi-component healthy lifestyle interventions. These interventions should focus on nutrition education and behavior change, tailored to meet the specific needs of the Saudi population.

## 2. Introduction

Child health is a multifaceted concern encompassing physical, mental, and social well-being, with nutrition playing a critical role in their overall development [1]. A balanced diet is essential for promoting normal physical growth and cognitive performance in children [2]. Establishing healthy dietary and lifestyle patterns during childhood is crucial to preventing both short- and long-term health conditions [3]. Undernourished children often experience a weaker immune system, stunted growth, and cognitive impairments, which can negatively affect their learning and academic performance [4]. Conversely, overnutrition is associated with psychological distress, low self-esteem, and a poorer quality of life [5]. In Saudi Arabia, however, many children face significant nutritional challenges, including obesity, malnutrition, and deficiencies in essential nutrients, which could adversely impact their health outcomes. Based on the use of World Health Organization (WHO) cut-offs to define obesity, the prevalence rates of underweight, overweight, and obesity among Saudi children are 17.2%, 19.1%, and 18.9%, respectively [6]. Both undernutrition and overnutrition can lead to adverse health outcomes [7]. Minimizing the risk of malnutrition should be prioritized to improve public health in Saudi Arabia.

Central to addressing these nutritional challenges is the concept of nutritional knowledge [8]. This knowledge refers to the awareness and understanding of dietary guidelines, food choices, and the impact of nutrition on health [9]. It has been reported that nutrition knowledge impacts food choices [10]. Gaining substantial nutritional knowledge is crucial for children, as it lays the groundwork for lifelong healthy eating habits. Understanding balanced diets, portion control, and the benefits of various food groups enables children to select foods wisely, which could promote their physical growth and mental development. Furthermore, this knowledge empowers children to resist unhealthy options, such as processed foods and sugary beverages, which could increase the risk of obesity and other health issues in the future [11,12] Enhancing the nutritional knowledge of schoolchildren could be considered an effective non-invasive tool to facilitate their nutritious food choices and dietary habits, thereby promoting their physical and mental health.

Several factors may significantly influence a child’s nutritional knowledge level and awareness. Additionally, nutritional knowledge and related factors may shape the understanding of healthy eating practices among children and influence their food choices. One essential factor is the guidance of parents, especially mothers [13]. Parents are key influencers in shaping children’s dietary habits and understanding the importance of balanced diets. Mothers who have a solid background and understanding of the critical role of nutrition might be in a better position to educate their children about healthy eating habits, which could enhance their health outcomes. Other studies have indicated that mothers with more nutritional knowledge are more likely to promote healthier dietary practices within their households, thereby positively promoting their children’s nutritional status [14]. For example, children whose parents exhibited healthier eating attitudes demonstrated stronger adherence to nutritious dietary patterns, which resulted in a lower likelihood of experiencing micronutrient deficiencies [14]. In contrast, children of mothers with lower educational levels and limited nutritional knowledge tended to have poor-quality diets [13]. These findings underscore the key role of maternal education and attitudes in influencing children’s nutritional outcomes.

In Saudi Arabia, there is a dearth of information concerning the extent of nutrition knowledge among schoolchildren and the determinants influencing this knowledge. Only one study targeting Saudi preschool children and their mothers found that the level of nutrition knowledge of mothers impacts their children’s dietary practices. For example, mothers with more nutrition knowledge of food additives tended to follow healthier behaviour and dietary practices better compared to mothers with a lower nutrition knowledge level [15]. Another study focused on Saudi adolescents reported that parents’ education level is positively associated with their adolescents’ nutrition knowledge [16]. Nonetheless, currently, no study has specifically investigated the level of nutrition knowledge among Saudi schoolchildren, nor has any research examined the influence of mothers’ nutrition knowledge on their children’s nutrition knowledge and their weight status. A focused analysis on the direct correlation between Saudi mothers’ nutrition knowledge and their schoolchildren’ knowledge and weight status remains absent. Hence, the nutrition knowledge of mothers in Saudi Arabia should be evaluated and improved since it could impact their children’s health.

This study sought to cover this gap in the literature by assessing the nutrition knowledge among Saudi schoolchildren and their mothers. Moreover, this study examined the association between the nutritional knowledge of mothers and the nutritional knowledge and weight status of their schoolchildren residing in Makkah City. Additionally, this study examined the factors associated with nutrition knowledge among these children. Identifying the determinants of higher nutrition knowledge scores among Saudi children and their mothers would enable stakeholders to tailor educational programs and address the specific needs of this group of the Saudi community. Thus, this could ultimately contribute to improving public health outcomes in Saudi Arabia.

## 3. Research Questions

This study aimed to address the following research questions: First, is there a positive association between the nutritional knowledge of Saudi mothers and that of their schoolchildren? Second, does the nutritional knowledge of Saudi mothers influence the weight status of their children? Finally, what sociodemographic factors are associated with the nutritional knowledge of schoolchildren in Saudi Arabia? These inquiries are essential for understanding the relationship between maternal nutritional knowledge and child health outcomes, as well as the broader implications for public health initiatives in the region.

## 4. Methods

### 4.1. Study Design and Participants

This cross-sectional study investigated the relationship between the nutritional knowledge of Saudi mothers and the weight status of their schoolchildren in Makkah City, Saudi Arabia. This study was conducted from 8 December 2023 to the end of February 2024. Recruitment involved open invitations through both online and in-person announcements, including flyer distribution in community centers and neighborhoods, as well as sharing information through school–parent groups and Umm Al-Qura University networks. Online invitations were primarily shared via school parents’ WhatsApp groups, community-based WhatsApp groups, and Umm Al-Qura University-affiliated networks. Then, interested mothers contacted the research team directly. A telephone screening was then arranged to assess eligibility by a trained research assistant, who confirmed the inclusion criteria and scheduled an appointment for eligible mother–child pairs. The mothers were the primary point of contact for recruitment. Those who agreed to participate provided written informed consent for both their own participation and that of their child(ren). All 100 children included in this study were the biological children of the 60 participating mothers. This study targeted children aged from 5 to 12 years. This age group represents a key window for preventive intervention, as unhealthy habits formed during childhood often track into adolescence and adulthood. By focusing on children aged from 5 to 12 years, this study captures a stage where maternal influence is still strong and where targeted nutrition education can have long-lasting effects on weight management and dietary choices.

Children were excluded if their mothers reported that the child had any chronic diseases (e.g., diabetes, cancer, and heart disease), eating disorders, or allergies (e.g., celiac disease). There were no specific inclusion or exclusion criteria for the mothers beyond the requirement that they were the mothers of the participating children.

### 4.2. Data Collection

#### 4.2.1. Mothers Questionnaire

Maternal sociodemographic information was obtained through an online questionnaire. Nutritional qualifications of the mothers were assessed by asking “Do you have any qualifications related to nutrition (or are you studying to obtain a nutrition-related qualification)?” The mothers’ nutritional knowledge was assessed using a validated Arabic version of the General Nutrition Knowledge Questionnaire (GNKQ) [17]. Given the cultural, dietary, and linguistic similarities across the Gulf countries, this tool was considered appropriate for the Saudi population. Briefly, the GNKQ consists of four sections that evaluate nutritional knowledge, including dietary recommendations, food groups and nutrient sources, healthy food choices, and associations between diet, diseases, and weight, with 86 questions in total. The questions were in a multiple-choice format, with the participants marking their responses. Responses were scored by awarding one point for each correct response and zero points for incorrect responses. The total nutrition knowledge score was calculated by summing the scores from the four sections.

#### 4.2.2. Children’s Knowledge and Practices

To ensure that the assessment covered a wide range of nutrition-related topics and provide a thorough evaluation of the participants’ important nutritional concepts, the children’s nutritional knowledge and practices were evaluated based on a set of 20 questions, which were derived from two different questionnaires [18,19]. The two questionnaires were completed through face-to-face interviews with the children. The questionnaire included two main sections: Section 1, Nutrition Knowledge (Questions 1–10). This section evaluated the child’s understanding of basic nutrition concepts, such as healthy and unhealthy foods, food groups, and the role of nutrients in the body. Each question required the child to select the correct statement among options (e.g., “True,” “False,” or “I don’t know”). Each of the 10 questions had a correct answer coded as 1 point and an incorrect answer coded as 0 points. Every child’s nutrition knowledge score was calculated by summing up the points earned across all 10 questions. Section 2, Nutrition-Related Habits and Practices (Questions 11–20). This section assessed dietary habits, such as consumption of fruits and vegetables, sweets, fast food, eating while watching TV, bringing lunch to school, and food preparation at home. Responses were recorded using a frequency scale (e.g., “Every day,” “Often,” “Sometimes,” “Rarely,” or “Never”).

#### 4.2.3. Anthropometric Measurements

For the children, three trained clinical nutrition students collected anthropometric measurements, including height, weight, and waist circumference, with duplicates for each included child using standardized protocols as per the WHO [20]. The height-for-age z-score (HAZ) and Body Mass Index (BMI)-for-age z-score (BAZ) were calculated using the WHO Anthro Plus software [21]. This software applies the 2007 WHO growth reference standards to derive the standardized z-scores, which reflect the child’s height and BMI relative to the expected values for their age and sex. The children were categorized into weight status groups based on the BMI z-scores using the 2007 WHO growth reference standard [22].

- Obesity: BMI-for-age z-score > +2 SD, equivalent to a BMI of 30 kg/m^2^ at 19 years of age;
- Overweight: BMI-for-age z-score > +1 standard deviation (SD), equivalent to a BMI of 25 kg/m^2^ at 19 years of age;
- Thinness: BMI-for-age z-score < −2 SD;
- Severe thinness: BMI-for-age z-score < −3 SD.

For the mothers, anthropometric data (weight and height) were self-reported via the questionnaire, and BMI was calculated using the standard formula: weight (kg)/height^2^ (m^2^). Ethical approval for this study was obtained from the UQU Faculty of Medicine, Scientific Research Ethics Committee (number: HAPO-02-K-012-2023-06-1695). Written informed consent was obtained from the mothers, and assent was obtained from the children before the commencement of this study.

#### 4.2.4. Sample Size

A priori sample size calculation was conducted using G*Power Version 3.1.9.6. A sample size of *n* = 98 was required to detect a medium effect size (f2 = 0.15) with 6 predictors in linear regression analysis and to achieve a power of 80% with an alpha error of 0.05 [23]. Due to the nature of this community-based recruitment, it was not feasible to determine the exact number of mothers approached for participation. However, a total of 60 mothers with 100 children were eligible and agreed to participate in this study.

#### 4.2.5. Statistical Analyses

The data from the mothers and their children were analyzed as follows: The continuous data are presented as the mean ± SD, while the categorical data are presented as frequencies (%). The data were analyzed using Stata version 14. Level of significance was set at (<0.05). The mean scores across BMI categories were assessed using ANOVA. Multiple linear regression analysis was used to examine the association between the mothers’ and children’s nutritional knowledge, adjusting for potential confounders.

## 5. Results

### 5.1. Sociodemographic Characteristics of Mothers and Children

This study included 100 children (51 girls and 49 boys) and their 60 mothers. Of the 60 mothers, 29 participated with one child, 29 participated with two children, 3 participated with three children, and 1 mother participated with four children. Table 1 shows the characteristics of the mothers (*n* = 60), and Table 2 shows the characteristics of the children (*n* = 100). Approximately half (51.67%) of the participating mothers (mothers) were in the age group of 30–40 years old, and almost (95%) all of them were married. Over half of the mothers held a bachelor’s degree and six (10%) had nutrition-related qualifications. The BMI was calculated, and 24 (40%) of the mothers had a normal weight. The remaining 36 mothers were equally divided into the overweight and obese groups. Simultaneously, the children’s BMI indicated that 66% of the participants were within the normal weight range, 15% were overweight, and 12% were classified as obese.

### 5.2. Mothers’ General Nutritional Knowledge Questionnaire (GNKQ)

Nutritional knowledge of the mothers was assessed using the GNKQ. The mean nutritional knowledge scores (48.32 ± 15.09). The mothers’ nutritional knowledge scores are shown in Table 3. Only two mothers (3.33%) have lots of nutritional knowledge, with the remaining (96%) having less nutritional knowledge.

### 5.3. Children’s Nutritional Knowledge Classifications and Eating Habits

Table 4 presents the classifications of nutritional knowledge alongside the consumption patterns of fast food, fruits, and vegetables. The results show that 54 children were identified as having less nutritional knowledge, 30 children had moderate/average nutritional knowledge, and only 16 had lots of nutritional knowledge. The eating habits that have been collected include fruit, vegetables, and fast-food consumption. The data indicate that 46 and 40% of the participants frequently consumed fruits and vegetables, respectively, at a rate of 4–5 times per week. Half of the participating children, however, consumed fast food from one to three times a week.

### 5.4. Relationship Between Maternal Nutritional Knowledge and Children’s Nutritional Knowledge and Weight Status

**Figure 1:**
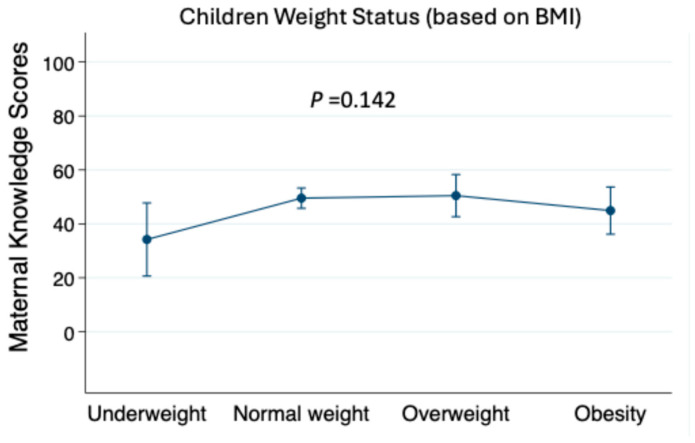
Maternal nutritional knowledge and children’s BMI classification. Data are presented as means, 95% confidence interval, and analyzed by one-way ANOVA.

**Figure 2:**
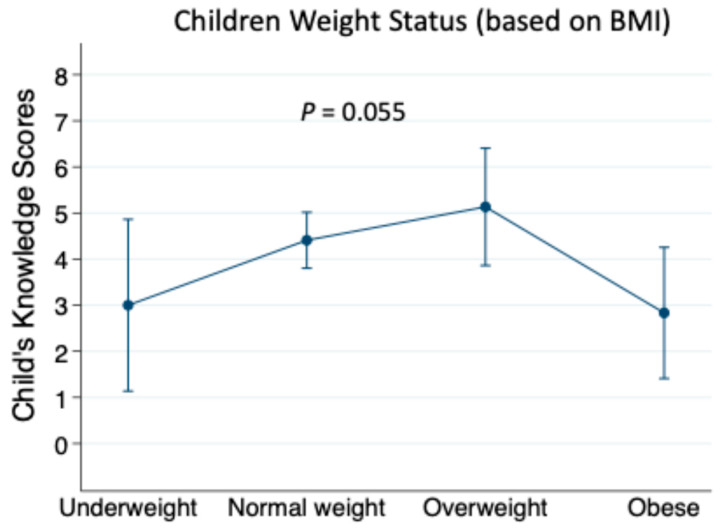
Children’s nutritional knowledge and children’s BMI classification. Data are presented as means, 95% confidence interval, and analyzed by one-way ANOVA.

To examine the association between the mothers’ knowledge and the children’s knowledge, we conducted multiple linear regression, adjusting for potential confounders, as shown in Table 5. The maternal nutritional knowledge scores were significantly associated with the children’s knowledge scores (β = 0.06, 95% CI: 0.03, 0.09; *p* < 0.001), suggesting that more maternal knowledge may contribute to greater nutritional awareness among children. This relationship remained significant after adjusting for the child’s age, sex, as well as maternal factors (age, marital status, education level, BMI, and whether she has any nutritional qualification). A significant positive association was observed between child’s age and their nutritional knowledge scores (β = 0.61, 95% CI: (0.47, 0.75); *p* < 0.001), indicating that the older children had higher knowledge scores. However, the sex differences in knowledge scores were not statistically significant (β = −0.52, 95% CI: −1.27, 0.24; *p* = 0.174). Among the maternal factors, age, educational level, marital status, having a nutritional qualification, and the BMI scores were not associated with the children’s knowledge scores. Additionally, Figure 1 illustrates one-way ANOVA analyses of maternal knowledge scores across the BMI categories for the children, showing a non-significant relationship (*p* = 0.142). The linear relationship was also not significant between the maternal knowledge scores and the child’s BMI z-scores using linear regression. Figure 2 presents the children’s nutritional knowledge scores across the BMI categories for the children, demonstrating a peak among the children of the overweight mothers, followed by a decline in the obese category, but this was a non-significant trend (*p* = 0.055). Post-hoc analysis was conducted with Bonferroni correction, but was also not significant.

## 6. Discussion

This study evaluated the nutritional knowledge and weight status of a sample of Saudi schoolchildren and their mothers. It found that overweight and obesity were prevalent, affecting over a quarter of the participating children and 60% of the mothers. Notably, only 16% of the participating children demonstrated a high level of nutrition knowledge, while 30% and 54% had moderate and low levels, respectively. With regards to the mothers, only 6.67% achieved a high level of nutrition knowledge, while 63.33% and 30% had moderate and low levels, respectively. Additionally, this study examined factors influencing nutritional knowledge among these children and identified a significant association between the nutritional knowledge of mothers and that of their children. These findings underscore the urgent need for targeted educational interventions for both mothers and children. Given the alarming rates of overweight and obesity in Saudi Arabia, it is essential to compare our findings with national statistics to assess the representativeness of this sample within the broader Saudi community.

Our results showed that 27% of the participating children were classified as having excess weight. Approximately one in four participating children experienced either overweight or obesity. These findings are consistent with the results of a recent national school screening program conducted between 2018 and 2022, which reported that 10.4%, 10.7%, and 4.5% of Saudi children aged from 6 to 19 years were classified as overweight, obese, and severely obese, respectively [24]. This also aligns with the findings of a large, multicenter, population-based study that included 351,195 children living in Saudi Arabia, which found that one-fifth of the participating children were either overweight or obese [25]. Moreover, this increase aligns with the global challenge of childhood obesity. A report published by The Lancet in 2025 projects that by 2050, approximately one in three children and young people worldwide will be affected by obesity [26]. These consistent trends highlight the critical need for effective public health interventions to combat this escalating epidemic. Within the context of Saudi Arabia, it is important to further investigate the factors that either facilitate or hinder participation in interventions aimed at improvement of nutrition knowledge and weight status among Saudi children.

This study reported an unsatisfactory level of nutrition knowledge among children, with 54% of them possessing low-level nutritional knowledge. The paucity of relevant studies examining the nutrition knowledge of young children in Saudi Arabia hinders meaningful comparisons with the current findings. Nevertheless, the observed low level of nutritional knowledge among the study participants is comparable to the results of a previous investigation conducted in a neighboring country, the United Arab Emirates (UAE). This earlier investigation assessed the nutritional knowledge of children aged from 9 to 13 years from four different schools and revealed that 86% of the participants had poor nutritional knowledge [27]. The level of nutritional knowledge was low not only among young Arab children, but also among adolescents. A large-scale survey conducted with 5401 adolescents across ten Arab countries indicated that more than one-quarter of the participants exhibited poor nutritional literacy [28]. In response to these challenges, the Ministry of Education in Saudi Arabia launched a recent initiative in 2025 aimed at delivering over 8 million healthy meals to approximately 212,000 students in schools across Riyadh, Jeddah, and Al-Ahsa [29]. It is important, however, to note that while nutritional information is incorporated into the school curriculum, there are currently no dedicated, regular courses focused exclusively on nutrition education. This situation underscores a significant opportunity for development within the educational framework, highlighting the need for structured nutrition education to improve dietary awareness among children.

The present study found that 30% of the mothers were classified as having a low level of nutrition knowledge, while 63.33% exhibited a moderate level, and only 6.67% demonstrated a high level of nutrition knowledge. This finding is consistent with the findings from a recent cross-sectional study that included 1845 Saudi parents of children aged 10–19 years focused on food literacy [16]. Bookari (2023) [16] found that 46% of parents from the Riyadh, Northern Borders, Jawf, and Hail regions had inadequate nutrition knowledge. It is essential, however, to note that Bookari’s study used the Short Food Literacy Questionnaire (SFLQ) to measure nutrition knowledge, while ours used the GNKQ. A recent study utilized the GNKQ, similar to our study, to assess the nutritional knowledge of 281 working mothers in Malaysia, reporting similar findings, with 76.16% of mothers demonstrating low-level nutritional knowledge [30]. Further, consistent findings were reported in a study assessing mothers of preschool children and their knowledge about food additives. The results indicated that 20.7% had poor knowledge, 32.6% had a moderate level of knowledge, and 46.6% had a high level of knowledge [15].

There are some factors that may influence a child’s nutritional knowledge level and awareness, which involve the guidance of parents, especially mothers [13]. Parents are key influencers in shaping children’s dietary habits and understanding the importance of balanced diets. Mothers who have a solid background and understanding of the critical role of nutrition might be in a better position to educate their children about healthy eating habits, which could enhance their health outcomes. Reflecting on the impact of mothers’ awareness and their nutritional knowledge, this study found a statistically significant positive correlation between mothers’ nutrition knowledge scores and the corresponding higher levels of nutrition knowledge in their children. This finding implies that Saudi mothers with greater nutritional awareness are able to positively impact the nutritional knowledge of their children. Moreover, several studies indicate that the dynamic interplay between mothers’ nutrition knowledge level and their own dietary habits is associated with corresponding health outcomes for their children [23,24,25]. A cross-sectional study concerning parental health literacy and nutrition literacy found that higher levels of nutrition literacy among parents of schoolchildren were associated with better feeding practices, such as increased monitoring of their children’s diet and avoidance of using food as a reward [31]. In addition, a systematic review of 15 studies confirmed that nutrition education programs targeting mothers are effective, positively influencing children’s health outcomes. These programs employed various strategies, including lectures, demonstrations, and brainstorming sessions [32].

It is important to note that despite the significant association detected between the nutritional knowledge of mothers and that of their children in this study, the analysis revealed no significant association between these mothers’ nutritional knowledge and their children’s BMI. The absence of this association may be due to the small sample size, potentially limiting the statistical power of the results. However, interestingly, a borderline significant association was identified between the children’s nutritional knowledge and their BMI, particularly among the overweight children. This suggests that those with a higher BMI may possess greater nutritional knowledge. It may indicate that children with overweight have a better understanding of nutrition, yet they still struggle with weight management. One potential explanation is that mothers of overweight children recognize this issue as critical, which motivates them to seek additional information regarding nutrition. Such increased awareness may subsequently enhance their children’s understanding of healthy eating practices. This observation underscores the notion that nutritional knowledge alone may not be sufficient for achieving a healthy weight status, especially considering the struggle with an obesogenic environment [33]. Therefore, it is important to develop multi-component interventions that focus on improving nutrition knowledge, while also encouraging physical activity and healthy eating. Additionally, it is essential to revise policies related to food and beverage options within school environments [34]. 

Implementing behavior change techniques as a critical aspect of multi-component interventions could be vital in helping children translate their nutritional knowledge into practice, fostering healthier attitudes and eating habits, which ultimately helps in weight management [35]. Implementing multi-component interventions that target several aspects of a healthy lifestyle could serve as an effective strategy for tackling childhood obesity. This claim is supported by a systematic review and meta-analysis of 19 randomized controlled trials (RCTs) that compared the effectiveness of different healthy lifestyle interventions for weight management among 58,649 children. The superiority of multi-component intervention over bi-component and single-component interventions has been statistically confirmed. Additionally, this study found that children with a higher initial BMI and body fat levels were more responsive to these lifestyle interventions, indicating that those who are overweight tend to benefit more from comprehensive lifestyle modifications [36]. This aligns with the findings of the present study, which demonstrated a borderline association between nutrition knowledge and BMI scores of overweight children, highlighting that individuals are typically more motivated to address issues when they are aware of a problem, rather than in the absence of such challenges.

Identifying suitable methods for delivering interventions is essential to guarantee their accessibility and effectiveness across a diverse audience. The use of interactive digital platforms may present a promising approach for delivering targeted interventions within the Saudi Arabian context. One such platform that has gained widespread adoption in Saudi Arabia is the messaging application, WhatsApp. In 2024, WhatsApp boasted the largest user base among Saudi residents, with approximately 83% of the population, or 21.5 million individuals, actively using the application [37]. WhatsApp is an effective tool for delivering interventions targeting parents of Saudi children with autism [38]. This underscores the potential for WhatsApp and similar digital platforms to serve as cost-effective and accessible channels for disseminating nutrition education and other health-related interventions within the Saudi context. Factors such as user engagement, behavior change techniques, and cultural adaptations should be investigated to develop evidence-based guidelines for utilizing WhatsApp as a sustainable and scalable intervention delivery method. Further research is needed to implement strategies that can optimize the effectiveness of digital platform-based nutrition interventions to improve health outcomes for the Saudi population.

With regards to the impact of sociodemographic factors on child nutritional knowledge, this study found a significant positive association between children’s age and their nutritional knowledge, with older children demonstrating better understanding compared to their younger counterparts. This aligns with the existing literature indicating that as children grow older, they are exposed to more nutrition-related education and experiences, which enhances their understanding of nutritional concepts [39]. This finding emphasizes the necessity for age-appropriate nutrition education that evolves alongside the child’s cognitive and developmental stages.

This study highlighted the essential role of mothers’ nutritional knowledge and its impact on their children’s level of knowledge. The findings of this study emphasized the importance of considering multi-component healthy lifestyle interventions that target children and their mothers to improve children’s health outcomes, particularly through age-appropriate nutrition education that aligns with children’s cognitive and developmental growth. In addition to assessing the nutritional knowledge of mothers, future studies are recommended to assess the nutritional knowledge of fathers as caregivers. Furthermore, future studies are needed to assess the nutritional knowledge of children and their caregivers in other regions in Saudi Arabia, considering the cultural differences among different regions. Examining the sex differences among a larger sample is recommended to generalize the findings.

### Strengths, Limitations and Recommendations

This study has strengths related to the discussed topic and methodology. Primarily, this was the first study to investigate the association between the nutrition knowledge of mothers and the corresponding nutrition knowledge levels of their children in Makkah City in Saudi Arabia. Additionally, the collected data employed in this study demonstrated a high level of accuracy, as the anthropometric measurements were obtained by trained clinical nutrition students’ adherence to the WHO criteria, rather than relying on self-reported data. Furthermore, the participants were engaged in a face-to-face individual interview to increase the accuracy and reliability of the collected data. Moreover, all the utilized questionnaires were validated instruments with clear scoring protocols, which facilitated meaningful comparisons to other relevant studies.

There are limitations to this study that should be acknowledged. Firstly, a limited sample size can affect the statistical power of analysis, making it difficult to detect meaningful relationships between variables. Consequently, the results may not accurately represent the broader population of children in Saudi Arabia. Future studies should recruit a larger sample size and employ more advanced statistical techniques to explore the relationships between variables. Additionally, the geographic restriction to Makkah City further impacts the generalizability of the findings. The demographic homogeneity of the participant pool limits the applicability of the results to children residing in other regions, where cultural, socioeconomic, and environmental factors may differ significantly. It is recommended that future studies collect data from all 13 provinces in Saudi Arabia to provide more representative findings.

Furthermore, the sample in this study was predominantly composed of mothers with a high school education or higher, which may not be representative of the broader Saudi population. Future research should aim for a more diverse participant pool, including individuals with varying educational backgrounds, to ensure that findings reflect the wider population. This would enhance the overall relevance and applicability of this research and provide a more comprehensive understanding of nutrition knowledge among children in Saudi Arabia. Another limitation is that we collected data from multiple children per mother, which may have introduced a potential bias; the responses from one child could influence or reflect the knowledge and attitudes of the others, rather than providing independent assessments. Moreover, the analysis did not adjust for intra-family clustering, primarily due to the modest sample size and the exploratory nature of this study. Future research with larger and more statistically powered samples is recommended to employ analytical approaches—such as multilevel modeling or generalized estimating equations—that account for clustering effects and provide more robust estimates.

Additionally, several potential confounders that may influence the association between maternal and child nutritional knowledge and child weight status were not assessed in this study. These include socioeconomic status, encompassing family income and parental employment; cultural beliefs and practices that shape dietary choices; access to nutrition education resources; and parenting styles that impact children’s eating habits. Additionally, peer influence on food behaviors and media exposure related to food marketing may also play significant roles. Future research should address these factors to enhance our understanding of the dynamics affecting nutritional knowledge and weight status among children. Finally, this study used surveys to assess nutrition knowledge; however, to enhance the reliability of future research, it is recommended that objective measures, such as dietary records and direct observations, be employed alongside surveys. These methods would provide a more accurate assessment of actual dietary habits associated with the level of children’s nutrition knowledge and behaviors, ultimately informing more effective interventions.

## 7. Conclusions

This study underscores the inadequate levels of nutritional knowledge among Saudi schoolchildren and their mothers. It was demonstrated that there is a significant correlation between mothers’ knowledge with that of their children. Given that over half of the children and nearly a third of mothers exhibited low levels of nutritional knowledge, there is a need for targeted educational interventions. Policymakers are recommended to consider implementing comprehensive multi-component interventions that include nutrition education programs directed at both mothers and children, as well as promoting physical activity and facilitating behavior changes. Such programs could leverage community resources and digital platforms to improve engagement and accessibility. By prioritizing the enhancement of maternal nutritional knowledge via multi-component healthy lifestyle interventions, it is possible to promote healthier dietary practices within families. Thus, this could contribute to a reduction in the prevalence of obesity and malnutrition among children in Saudi Arabia.

## Figures and Tables

**Table 1:** Sociodemographic characteristics of mothers.

Parameter	Mothers (*n* = 60)
Age group	
21–30 years old	10 (16.67%)
31–40 years old	31 (51.67%)
41–50 years old	17 (28.33%)
>50 years old	2 (3.33%)
Marital Status	
Married	57 (95%)
Separated/divorced	3 (5%)
Education	
<Bachelor’s degree	18 (30%)
≥Bachelor’s degree	42 (70%)
Nutritional knowledge qualifications	
Yes	6 (10%)
No	54 (90%)
Perceived health status	
Excellent	14 (23.33%)
Very good	13 (21.67%)
Good	21 (35%)
Moderate	8 (13.33%)
Weak	4 (6.67%)
BMI classification ^1^	
Underweight	0
Normal weight	24 (40%)
Overweight	18 (30%)
Obesity	18 (30%)

Values are presented as n (%). ^1^ Body Mass Index (BMI) was classified as follows: underweight (BMI < 18.5 kg/m^2^), healthy (BMI: 18.5–24.9 kg/m^2^), overweight (25–29.9 kg/m^2^), and obesity (BMI ≥ 30 kg/m^2^).

**Table 2:** Sociodemographic characteristics of children.

Parameter	Children (*n* = 100)
Sex	
Female	51 (51%)
Male	49 (49%)
Age group	
5–6 years old	21 (21%)
7–9 years old	39 (39%)
10–12 years old	40 (40%)
BMI classification ^1^	
Underweight	7 (7%)
Normal weight	66 (66%)
Overweight	15 (15%)
Obesity	12 (12%)

Values are presented as n (%). ^1^ Body Mass Index (BMI) was classified as follows: underweight—BMI-for-age z-score < −2 SD normal weight, and BMI-for-age z-score of < +1 SD to < −2; overweight—BMI-for-age z-score > +1 SD; obesity—BMI-for-age z-score of > +2 SD.

**Table 3:** Mothers’ General Nutritional Knowledge Questionnaire scores.

Parameter	All (*n* = 60)	Mean Percent of Correct Answers
GNKQ score	48.32 ± 15.09	56.18 ± 17.55%
Nutritional knowledge categories ^1^		
High	4 (6.67%)	
Moderate	38 (63.33%)	
Low	18 (30.00%)	
GNKQ scores by GNKQ section		
Experts advise	12.32 ± 4.15	55.1 ± 19.1%
Food groups	17.55 ± 6.27	56.9 ± 20.2%
Food choices	5.32 ± 2.50	44.3 ± 20.8%
Health problems and weight management	13.13 ± 4.10	62.5 ± 19.5%

Values are presented as means ± SDs or n (%). ^1^ Knowledge score classification was as follows: high-level nutritional knowledge >75% of correct answers, moderate nutritional knowledge is from 51% to 75% of correct answers, and low-level nutritional knowledge ≤50% of correct answers.

**Table 4:** Children’s nutritional knowledge classification and eating habits.

Parameter	Children (*n* = 100)
Children knowledge score ^1^	4.23 ± 2.54
Low nutritional knowledge	54 (54%)
Moderate nutritional knowledge	30 (30%)
High nutritional knowledge	16 (16%)
Fast food consumption ^2^	
Never	3 (3%)
Sometimes	50 (50%)
Often	33 (33%)
Daily	7 (7%)
Rarely	7 (7%)
Fruit consumption ^2^	
Never	5 (5%)
Sometimes	24 (24%)
Often	46 (46%)
Daily	22 (22%)
Rarely	3 (3%)
Vegetable consumption ^2^	
Never	14 (14%)
Sometime	37 (37%)
Often	40 (40%)
Daily	6 (6%)
Rarely	3 (3%)

Values are presented as means ± SDs or n (%). ^1^ Knowledge scores are classified as follows: <40%, low score; 41–69%, moderate; and >70%, high. ^2^ Consumption is classified as follows: never, less than once or twice a month; rarely, once, or twice a month; sometimes, from one to three times a week; and often, four or five times a week.

**Table 5:** Determinants of nutritional knowledge among Saudi children.

Predictors	B (95% CI)	SE	T	Standardized Coefficient *β*
Children, age, years	0.61 (0.47, 0.75) ***	0.07	8.70	0.55
Children, sex (boys vs. girls)	−0.52 (−1.27, 0.24)	0.38	−1.38	−0.10
Mothers, age. > 30 vs. ≤30 years	0.51 (−0.54, 1.57)	0.53	0.98	0.08
Mothers, marital status (separated vs. married)	0.41 (−0.73, 1.55)	0.57	0.72	0.03
Mothers, education (≥Bachelor’s degree vs. <Bachelor’s degree)	0.01 (−1.02, 1.04)	0.51	0.02	0.00
Mothers, nutritional qualification (yes vs. no)	1.23 (−0.44, 2.91)	0.84	1.47	0.15
Mothers. BMI, kg/m^2^	−0.01 (−0.07, 0.04)	0.03	−0.42	−0.03
Mothers’ nutritional knowledge scores	0.06 (0.02, 0.09) ***	0.02	2.80	0.33

Multiple linear regression was used with children’s nutritional knowledge as an outcome (*n* = 97). Model’s R^2^ = 0.51; F_(8, 59)_ = 20.41 *** with root mean square error (MSE) of 1.88. *** *p* < 0.001.

## Data Availability

The data presented in this study are available on request from the corresponding author.
